# EphB2 stem-related and EphA2 progression-related miRNA-based networks in progressive stages of CRC evolution: clinical significance and potential miRNA drivers

**DOI:** 10.1186/s12943-018-0912-z

**Published:** 2018-11-30

**Authors:** Mariangela De Robertis, Tommaso Mazza, Caterina Fusilli, Luisa Loiacono, Maria Luana Poeta, Massimo Sanchez, Emanuela Massi, Giuseppe Lamorte, Maria Grazia Diodoro, Edoardo Pescarmona, Emanuela Signori, Graziano Pesole, Angelo Luigi Vescovi, Jesus Garcia-Foncillas, Vito Michele Fazio

**Affiliations:** 1 Laboratory of Molecular Medicine and Biotechnology, University Campus Bio-Medico of Rome, via Alvaro del Portillo 21, 00128 Rome, Italy; 2 Department of Biosciences, Biotechnology and Biopharmaceutics, University of Bari “A. Moro”, via Orabona 4, 70126 Bari, Italy; 3 Fondazione IRCCS Casa Sollievo della Sofferenza, Bioinformatics Unit, viale dei Cappuccini, 71013 San Giovanni Rotondo, FG Italy; 4 New Drug Modalities, Drug Safety and Metabolism, AstraZeneca iMED Biotech Unit, Cambridge, UK; 5 Core Facilities - Cytometry unit, Istituto Superiore di Sanità, Viale Regina Elena 299, 00161 Rome, Italy; 6 Fondazione IRCCS Casa Sollievo della Sofferenza, viale dei Cappuccini, 71013 San Giovanni Rotondo, FG Italy; 7 Department of Pathology, IRCCS “Regina Elena”, National Cancer Institute, Via E. Chianesi 53, 00144 Rome, Italy; 8 Laboratory of Molecular Pathology and Experimental Oncology, Institute of Translational Pharmacology, Consiglio Nazionale delle Ricerche (CNR), Via Fosso del Cavaliere 100, 00133 Rome, Italy; 9 Institute of Biomembranes, Bioenergetics and Molecular Biotechnologies, Consiglio Nazionale delle Ricerche (CNR), Via Amendola 165/A, 70126 Bari, Italy; 10 Cancer Institute, University Hospital “Fundacion Jimenez Diaz”, Autonomous University, Av. Reyes Catolicos 2, 28040 Madrid, Spain; 11 Fondazione IRCCS Casa Sollievo della Sofferenza, Laboratory of Oncology, viale dei Cappuccini, 71013 San Giovanni Rotondo, FG Italy

**Keywords:** Colorectal cancer, MicroRNA, EphA2 and EphB2, Cancer stem cells, Biomarkers

## Abstract

EphB2 and EphA2 control stemness and differentiation in the intestinal mucosa, but the way they cooperate with the complex mechanisms underlying tumor heterogeneity and how they affect the therapeutic outcome in colorectal cancer (CRC) patients, remain unclear. MicroRNA (miRNA) expression profiling along with pathway analysis provide comprehensive information on the dysregulation of multiple crucial pathways in CRC.

Through a network-based approach founded on the characterization of progressive miRNAomes centered on EphA2/EphB2 signaling during tumor development in the AOM/DSS murine model, we found a miRNA-dependent orchestration of EphB2-specific stem-like properties in earlier phases of colorectal tumorigenesis and the EphA2-specific control of tumor progression in the latest CRC phases. Furthermore, two transcriptional signatures that are specifically dependent on the EphA2/EphB2 signaling pathways were identified, namely EphA2, miR-423-5p, CREB1, ADAMTS14, and EphB2, miR-31-5p, mir-31-3p, CRK, CXCL12, ARPC5, SRC.

EphA2- and EphB2-related signatures were validated for their expression and clinical value in 1663 CRC patients. In multivariate analysis, both signatures were predictive of survival and tumor progression.

The early dysregulation of miRs-31, as observed in the murine samples, was also confirmed on 49 human tissue samples including preneoplastic lesions and tumors. In light of these findings, miRs-31 emerged as novel potential drivers of CRC initiation.

Our study evidenced a miRNA-dependent orchestration of EphB2 stem-related networks at the onset and EphA2-related cancer-progression networks in advanced stages of CRC evolution, suggesting new predictive biomarkers and potential therapeutic targets.

**Electronic supplementary material:**

The online version of this article (10.1186/s12943-018-0912-z) contains supplementary material, which is available to authorized users.

EphA2 and EphB2 are tyrosine kinase receptors that are involved in complex biochemical mechanisms underlying tumor heterogeneity primarily by guiding the positioning of intestinal cells with distinct stemness or differentiation properties [1]. EphA2_high_ cells are localized in the upper differentiated crypt region, while EphB2 is expressed in a decreasing gradient from the crypt base toward the upper cell compartment and corresponds to an intestinal stem cell (ISC) marker [2]. At the onset of colorectal cancer (CRC), early events trigger continuous stem-like self-renewing state, giving rise to adenoma, while additional driver pathway alterations confer invasive behavior in advanced carcinoma [3]. EphA2 and EphB2 undergo progressive dysregulation in carcinogenesis that may resemble these different stages, so representing attractive druggable targets [4].

MicroRNA (miRNA) redundancy and the capacity of individual miRNAs to simultaneously regulate large cohorts of genes have shaped by evolution combinatorial miRNA-target networks that profoundly affect cellular properties, including the promotion of tumorigenic processes [5]. A network-based approach founded on the characterization of progressive miRNAomes centered on EphA2/EphB2 signaling during tumor development may unveil important regulatory networks and oncogenic targets.

Given that EphA2 and EphB2 have opposite distribution and roles along the intestinal crypt, we hypothesized that these receptors may influence alternative cell behaviors on initial or advanced CRC stages. Through an integrative translational approach (Additional File 1: Figure S1) and a miRNAome-guided pathway analysis, we defined two transcriptional signatures that are associated with EphB2 cells/early CRC phases and EphA2 cells/late CRC phases, with significant prognostic value.

## Results and discussion

### Distinct miRNAs characterize progressive stages of CRC development

**Fig. 1:**
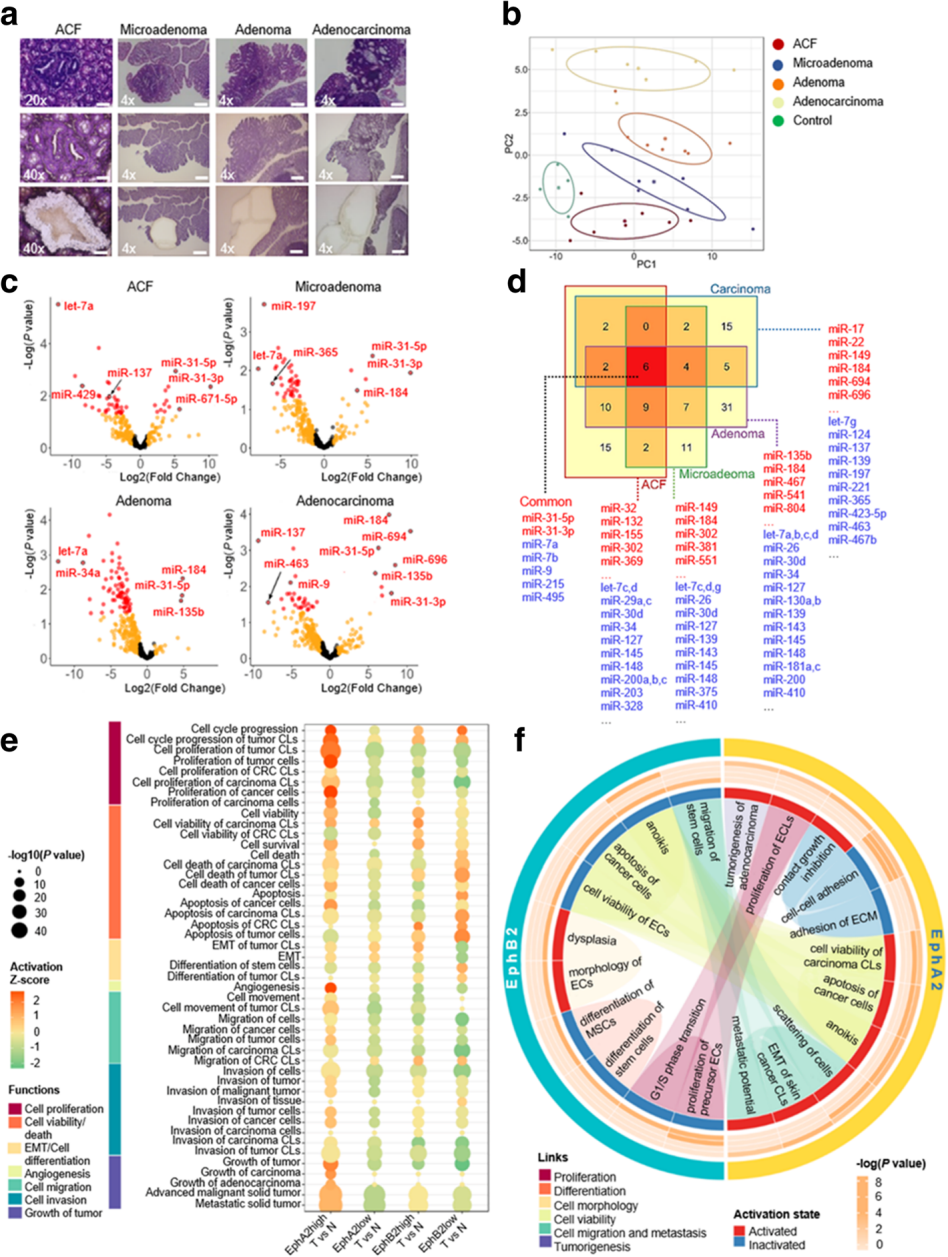
MiRNA expression profiling and functional enrichment analysis in progressive stages of murine CRC development: **a** Laser capture microdissection (LCM) of pretumoral lesions and tumors. First row, standard hematoxylin-eosin staining; second row, samples before LCM; third row, samples after LCM. Original magnification × 4 (scale bar 200 μm), × 20 (scale bar 50 μm), or × 40 (scale bar 20 μm). **b** Clustering of phases and control samples based on the first two principal components (PC1 and PC2) in a p partial least square - discriminant analysis (sPLS-DA) model. Control: normal colon mucosa of untreated mice. **c** Volcano plots of differentially expressed (DE) miRNAs (*P* value < 0.05 and |fold change| > 2) (red dots), miRNAs with only |fold change| > 2 (orange dots), and other miRNAs (black dots). The results were obtained through comparisons between each phase and control samples. **d** Venn diagram representing the number of miRNAs altered in each phase and shared between different phases (numbers in Venn diagram) of CRC. Sets of well-known CRC-associated miRNAs are indicated for each CRC phase (blue: down-regulated; red: up-regulated). **e** Enriched biological functions regulated by the DE miRNAs for Tumor (T) vs. Normal (N) of EphA2- and EphB2-positive cell comparisons. EphA2_high_ tumor cells are significantly enriched in activated functions related to *cell proliferation, cell viability* and *cell death*, *angiogenesis*, *cell migration*, *cell invasion* and *growth of tumor* (*P* < 0.0001). EphB2_high_ tumor cells show instead a significant enrichment of functions related to *cell viability* and a decreased or no activation of *cell death/apoptosis* (*P* < 0.0001). *EMT* activation and *cell differentiation* inactivation are also observed in EphB2_high_ tumor cells, a behavior coherently inverted in EphB2_low_ tumor cells (*P* < 0.0001). Sizes of bubbles are proportional to the statistical significance of the enriched functions (Fisher’s exact test). Gradients of color vary from red (activation) to green (inhibition) according to the Z-scores calculated for each enriched function and for each comparison. Functions are grouped into macro-categories. **f** Biological functions regulated by the DE miRNAs (and their target genes) with concordant fold changes between EphB2_high_ cells and tissues and EphA2_high_ cells and tissues. The outer circle marks the biological functions that are enriched in the EphA2_high_ and EphB2_high_ cells. The heatmap represents -log(*P* value) for each biological function evaluated in the four tissue types of adenocarcinoma (outer), adenoma, microadenoma, and ACF (inner). Blue and red rectangles represent the predicted activation and inactivation, respectively, of biological functions. These rectangles are linked by colored edges according to the macro-categories they belong to (proliferation, differentiation, cell morphology, etc.). Statistical significance was calculated using Fisher’s exact test as implemented in IPA. Abbreviations: CLs, cell lines; CRC, colorectal cancer; ECs, epithelial cells; ECLs, EC lines; ECM, extracellular matrix; EMT, epithelial-mesenchymal transition; MSCs, mesenchymal stem cells

Given the poorly foreseeable multiphase pattern of human CRC (hCRC) development, we used the well-characterized azoxymethane (AOM)/dextran sodium sulfate (DSS) murine model of sporadic CRC to obtain a predictable neoplastic evolution through the colorectal “Aberrant crypt foci (ACF)-microadenoma-adenoma-carcinoma” sequence, where each phase was expected to most likely progress to the following one [6, 7] (Fig. 1a; Additional file 1: Figure S2).

Of 641 miRNAs analyzed by TaqMan Low-Density Array (TLDA), 121 miRNAs were differentially expressed in the four murine CRC phases relative to normal mucosa (*P* < 0.05) (Fig. 1b, c, and d; Additional file 1: Figures S3 and S4). We found that the AOM/DSS model not only recapitulates several genetic modifications of hCRC [6], but also showed the aberrant expression of miRNAs belonging to hCRC-related pathways. In particular, miR-135b, targeting APC/β-catenin pathway [8], was up-regulated in murine adenoma (*P* < 0.05); let-7 family and miR-143, acting as tumor-suppressors in KRAS-driven CRC [9], were down-regulated already in ACF (let-7a, c, d, f *P* < 0.05) and in microadenoma (miR-143 *P* < 0.005); miR-9 was down-regulated in all the stages (*P* < 0.05), correlating with its tumor-suppressor function in hCRC by mediating invasion and metastasis [10] (Fig. 1c, d). More importantly, miR-31-5p and miR-31-3p were the most up-regulated in the earliest phases (*P* < 0.05 in ACF) (Fig. 1c, d), suggesting that these miRNAs may be involved in the CRC initiation.

### Distinct EphA2/EphB2-specific miRNAomes orchestrate cell proliferation/stemness/migration in progressive murine CRC stages

Based on cytofluorimetric, immunohistochemical and molecular characterization (Additional file 2: Methods and Materials) of EphA2_high/low_ and EphB2_high/low_ cells isolated from murine adenocarcinoma and normal colon (Additional file 1: Figure S5), we ascertained that EphA2 and EphB2 mark murine tumor cell subpopulations bearing either a differentiated or a stem-like phenotype, respectively.

With this assumption, we performed a TLDA-based miRNA expression analysis to characterize miRNAomes related to EphA2_high/low_ and EphB2_high/low_ CRC cells (Additional file 3: Tables S1, S2).

A list of 180 miRNAs was associated with EphA2 and CRC. Moreover, according to the EphB2_high_ CRC cells well-accepted identity of putative CSCs [2], we found they were enriched with miRNAs dysregulated in colon CSCs, such as miR-137, miR-34a, miR-215, miR-328 and miR-203 (*P* < 0.0001) [11].

Figure 1e describes a functional enrichment of the EphA2/EphB2 differential miRNAomes. All the functions analyzed were principally under control of miRNA targets listed in Additional file 3: Table S3.

When compared with the miRNAomes of murine ACF, microadenoma, adenoma and adenocarcinoma, the EphA2 and EphB2 tumor cells’ miRNAomes resulted in the alternate control of specific functions in different CRC stages (Fig. 1f; Additional file 3: Table S4).

It is worth noting that functions concerning *cell morphology/cell differentiation* and the inactivation of *cell proliferation* and *cell survival/death* were exclusively associated with EphB2 and enriched in ACF. In contrast, *cell survival/death* and *cell proliferation*, *cell migration and metastasis* and the reduction of *cell adhesion* were significantly associated with EphA2 and enriched in adenoma and adenocarcinoma.

These results indicate that while ACF might be enriched with EphB2_high_ tumor-initiating cells, which exhibit driver-gene/miRNA influencing CRC initiation via stem-related functions, the latest stages of murine CRC show an enrichment with EphA2_high_ cells orchestrating functions primarily related to tumor malignancy.

### Establishment of two CRC EphA2/EphB2-specific signatures

**Fig. 2:**
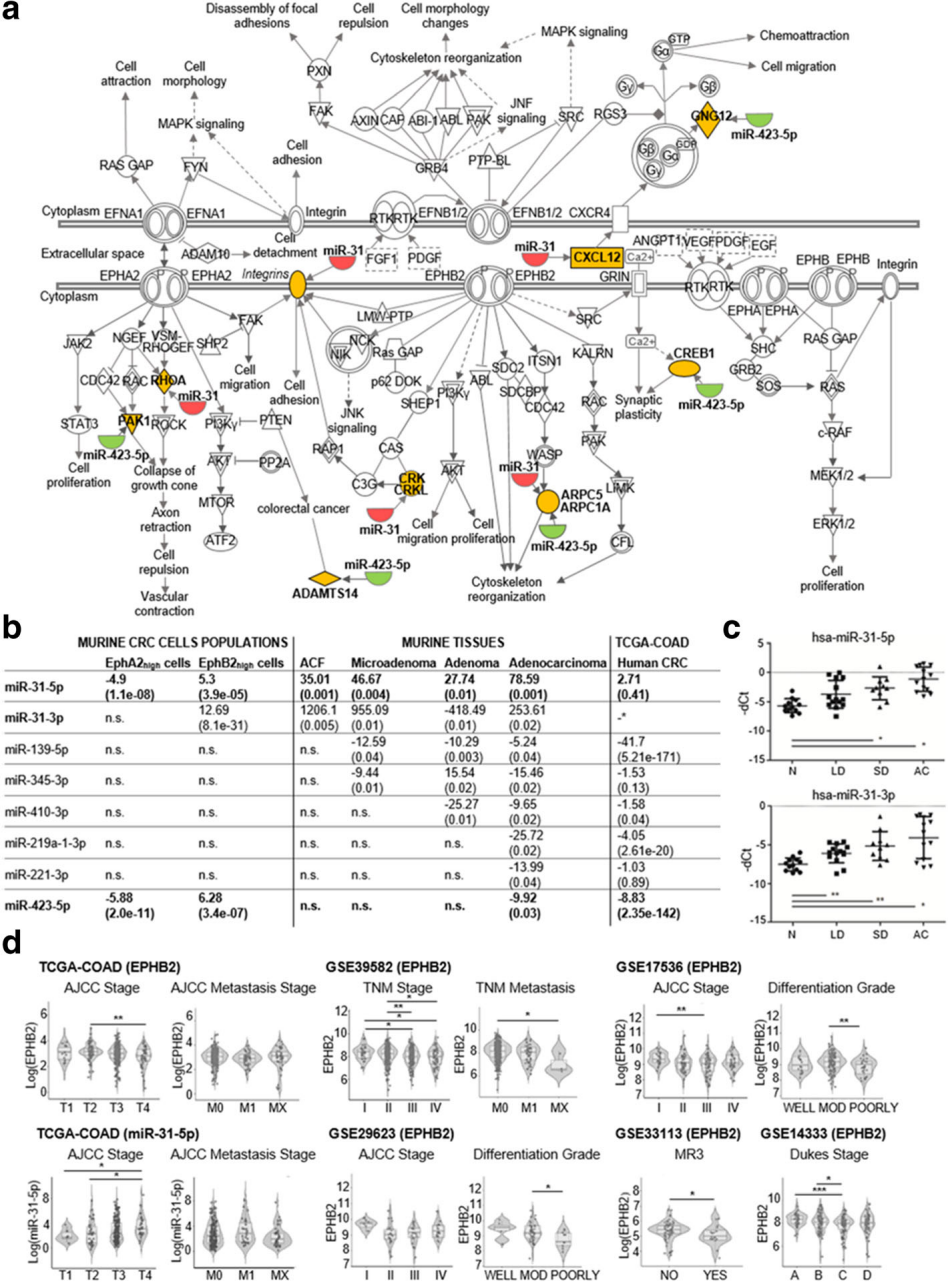
**a** Scheme of a macro-pathway including both the EphA2 and EphB2 pathways with their cross-talk links. Down-regulated and up-regulated miRNAs (miRs-31 and miR-423-5p) and their mRNA targets are colored in green, red and yellow respectively. **b** Summary table of Fold Change data with correlated *P* values shown in brackets of 8 Eph pathway-related miRNAs concordantly DE in human CRC (TCGA-COAD) and in both EphA2 or EphB2 murine CRC cells. In bold the miRNAs concordantly DE in hCRC and in both murine EphA2/EphB2 cells and tissues. *no -3p and -5p specification in miRNA nomenclature. Abbreviations: n.s., not significant. **c** qPCR analysis of miR-31-5p and miR-31-3p expression in normal colon mucosa (N), colorectal adenoma with low dysplasia (LD), colorectal adenoma with high dysplasia (HD), and adenocarcinoma (AC) human samples. Data are represented as the means +/− standard deviation (SD). Data are normalized by geometric mean of *RNU48* and *RNU6B*. Statistically significant differences were calculated using Student’s *t*-test. *P < 0.05, **P < 0.01. **d** EphB2 and miR-31-5p analysis on hCRC staging progression (AJCC stage, TNM stage, Dukes stage), metastasis, and differentiation grade. Dots indicate individual data points, and their distribution is represented by violin and box plots. The box plots show the interquartile range and median value of the distribution. Abbreviations: FC, fold change; MOD, moderately differentiated; MR3, metastasis or recurrence within 3 years. *P < 0.05; **P < 0.01 and ***P < 0.001; Mann-Whitney-U test

Considering the EphA2/EphB2 macro-pathway (Fig. 2a), we compared Eph pathway-related miRNAs included in our preclinical results to those dysregulated in CRC patients (The Cancer Genome Atlas - colon adenocarcinoma, TCGA-COAD). Eight miRNAs were concordantly dysregulated in both murine and human CRC (Fig. 2b). Strikingly, among them, miR-31-5p and miR-31-3p were up-regulated in all four murine CRC stages and in the EphB2_high_ tumor cells. Conversely, miR-423-5p was down-regulated in the advanced murine CRC phases and in the EphA2_high_ tumor cells. These miRNAs displayed inverse regulation in the two cell subpopulations (Fig. 2b), supporting the strong association of miRs-31 and miR-423-5p not only with specific CRC phases but also with specific Eph receptor-expressing cells.

The pathway analysis of 617 genes target of miRs-31 and miR-423-5p (Additional file 3: Tables S5 and S6) resulted to significantly enrich CRC-related signaling pathways (*P* value < 0.05) (Additional file 1: Figures S6 and S7).

On that basis, we defined two EphA2-/EphB2-related signatures by the combination of miRs-31 and miR-423-5p with their experimentally validated target genes belonging to the EphA2/EphB2 macro-pathway. Therefore, the EphB2 signature comprehended miRs-31, *ROHA*, *CRKL*, *CRK*, *ITGA5*, *CXCL12*, *ARPC5*, *RASA1* and *SRC*, while the EphA2 signature was composed of miR-423-5p, *ARPC1A*, *PAK1*, *GNG12*, *CREB1* and *ADAMTS14*.

### Up-regulation of miRs-31 occurs at very early stages of hCRC development

Given the importance of the early dysregulation of both miR-31-5p and miR-31-3p in the murine CRC, for their possible involvement in driving tumor initiation, we focused on the validation of their expression levels in the earliest stages of hCRC. We used a selected set of 49 human samples including low dysplasia (LD)-adenoma (*n* = 14), severe dysplasia (SD)-adenoma (*n* = 10), adenocarcinoma (*n* = 13) and normal mucosa (*n* = 12), that are not available in public datasets. qPCR analysis confirmed a significant overexpression of both miRs-31 in all the samples with an upward trend from LD-adenoma (preneoplastic) to SD-adenoma and adenocarcinoma, relative to normal mucosa (Student’s *t*-test, *P <* 0.05) (Fig. 2c). This is in line with recent demonstrations of miR-31-5p acting as a master modulator of the ISCs niche signaling during normal homeostasis, regeneration and tumorigenesis [12] or as potential driver of lung cancer initiation via KRAS pathway [13].

### Prognostic value of EphA2/EphB2-specific signatures

Both EphA2 and EphB2 signatures were validated in 1663 CRC patients from nine cohorts of TCGA-COAD and Gene Expression Omnibus (GEO) databases, through a differential expression analysis between tumor and normal samples (Additional file 3: Table S7; Table 1a). *EPHA2* was overexpressed in different datasets (*P* < 0.02) and miR-423-5p was concomitantly down-regulated (*P* < 0.01), whereas *CREB1* and *ADAMTS14* were overexpressed (*P* < 0.05). However, no significant changes in the expression levels of both *EPHA2* and miR-423-5p were observed when they were analyzed in correlation with tumor staging progression (Additional file 1: Figure S8). *EPHB2* was significantly overexpressed in TCGA-COAD (*P* = 0.03), GSE35834 cohort (*P* = 0.0008) and in patients with colorectal adenoma of GSE4183 cohort (*P* < 0.001); miR-31-5p was also overexpressed (*P* < 0.03) and its targets *CRK*, *CXCL12*, *ARPC5* and *SRC* were down-regulated in three out of six cohorts. It is worth noting that in the analysis of the GSE4183 cohort, the expression level of EphB2 decreased in the transition from adenoma to carcinoma. In line with this observation, we found that *EPHB2* expression levels gradually decreased with the progression of the TNM/AJCC stage and with metastasis (Fig. 2d). These results support the model that during CRC progression, stem EphB2-dependent functions are confined to a very small cell fraction of the tumor mass [7, 14], which likely represents the tumorigenic cell reservoir. Moreover, a gradual increase of miR-31-5p expression levels was observed with the progression of the TNM/AJCC stage (Fig. 2d), suggesting that it could be associated not only with particular miRNA patterns of stem-like EphB2_high_ cells during tumorigenesis, but also with miRNA patterns of different cell populations replenishing the tumor mass in progressive CRC stages. Our findings are in line with functional studies showing that miR-31 has pleiotropic activity and is typically overexpressed with high expression correlating with advanced CRC disease [15].

**Table 1:** Validation of the EphA2 and EphB2 signatures in GEO and TCGA-COAD public databases

**a.**						
	**GSE35982** FC (*P* value)	**GSE35834** FC (*P* value)	**TCGA-COAD** FC (*P* value)	**GSE4183**		
				**Adenoma** FC (*P* value)	**Carcinoma** FC (*P* value)	**IBD** FC (*P* value)
**EPHA2**	-1.16 (0.35)	**1.18** **(0.02)**	1.27 (0.40)	1.7 (0.07)	1.67 (0.08)	**2.16** **(0.003)**
**miR-423-5p**	-2.2 (0.30)	-1.26 (0.09)	**-8.82** **(2.35e-142)**	-	-	-
ARPC1A	-1.07 (0.20)	1.12 (0.17)	-1.09 (0.35)	1.14 (0.32)	1.16 (0.24)	-1.13 (0.39)
PAK1	-1.01 (0.94)	-1.07 (0.25)	-1.21 (0.01)	-1.27 (0.02)	-1.49 (0.0006)	-1.63 (7.42e-05)
GNG12	1.11 (0.46)	-1.63 (2.48e-08)	-1 (0.128)	-1.44 (0.005)	-1.28 (0.04)	-1.35 (0.02)
**CREB1**	-1.06 (0.40)	-1.04 (0.38)	**2.72** **(2.85e-34)**	1.16 (0.15)	**1.36** **(0.005)**	**1.26** **(0.04)**
**ADAMTS14**	-1.03 (0.67)	1.03 (0.52)	**2.81** **(0.0003)**	-1.05 (0.85)	1.32 (0.21)	**1.52** **(0.05)**
**EPHB2**	1.19 (0.49)	**1.33** **(0.0008)**	**1.75** **(0.03)**	**3.39** **(7.75e-06)**	1.26 (0.16)	-1.13 (0.67)
**miR-31-5p**	**49.65** **(0.02)**	**3.17** **(0.03)**	2.71 (0.41)	-	-	-
**miR-31-3p**	**14.73** **(0.005)**	-	-	-	-	-
RHOA	1.10 (0.08)	-1.01 (0.71)	-1.08 (0.28)	1.12 (0.06)	-1.29 (0.21)	1.09 (0.13)
CRKL	1.07 (0.54)	1.12 (0.002)	1.18 (0.03)	1.20 (0.139)	1.13 (0.14)	-1.19 (0.22)
**CRK**	-1.11 (0.36)	**-1.25** **(0.0008)**	-1.12 (0.10)	**-1.22** **(0.02)**	-1.15 (0.09)	-1.13 (0.12)
ITGA5	1.78 (0.30)	-1.08 (0.38)	-1.13 (0.64)	1.88 (0.59)	4.50 (0.03)	4.56 (0.03)
**CXCL12**	-1.19 (0.69)	**-2.40** **(9.44e-14)**	**-3.00** **(6.55e-18)**	**-6.91** **(9.80E-09)**	**-2.90** **(2.95E-06)**	**-1.48** **(0.01)**
**ARPC5**	**-1.18** **(0.05)**	1.01 (0.62)	1.09 (0.36)	-1.33 (0.06)	**-1.36** **(0.05)**	**-1.37** **(0.05)**
RASA1	-1.03 (0.61)	1.19 (0.04)	2.60 (1.92e-20)	1.21 (0.02)	1.11 (0.23)	-1.10 (0.30)
**SRC**	1.23 (0.16)	1.20 (0.0008)	**-1.37** **(0.02)**	-1.37 (0.10)	**-1.57** **(0.03)**	1.79 (0.03)
**b.**						
**Multivariate analysis of EPHA2 genetic signature without hsa-miR-423-5p**						
		**Relative Risk**		**95% CI**	***P*** **value**	
**TCGA-COAD (OS)**						
ADAMTS14		1.07		0.97 to 1.19	0.17	
CREB1		0.97		0.87 to 1.09	0.65	
**GSE33113 (MR3)**						
ADAMTS14		1.03		0.99 to 1.06	**0.05**	
CREB1		1.01		0.99 to 1.03	0.44	
**GSE14333 (DFS)**						
ADAMTS14		0.76		0.65 to 0.90	**0.001**	
CREB1		0.99		0.54 to 1.84	0.99	
**GSE17536 (OS)**						
ADAMTS14		0.79		0.26 to 2.45	0.69	
CREB1		1.43		0.59 to 3.48	0.44	
**GSE29623 (OS)**						
ADAMTS14		0.36		0.04 to 3.06	0.36	
CREB1		0.82		0.13 to 5.1	0.83	
**Multivariate analysis of EPHB2 genetic signature without hsa-miR-31-5p**						
		**Relative Risk**		**95% CI**	***P*** **value**	
**TCGA-COAD (OS)**						
CRK		0.97		0.94 to 1.01	0.15	
CXCL12		1.03		0.99 to 1.07	0.17	
ARPC5		0.99		0.96 to 1.02	0.53	
SRC		1.00		0.97 to 1.02	0.79	
**GSE33113 (MR3)**						
CRK		1.01		1.00 to 1.01	**0.01**	
CXCL12		1.00		1.00 to 1.00	**0.02**	
ARPC5		1.00		1.00 to 1.01	**0.05**	
SRC		1.00		0.95 to 1.05	0.95	
**GSE14333 (DFS)**						
CRK		0.80		0.54 to 1.20	0.29	
CXCL12		0.74		0.60 to 0.92	**0.01**	
ARPC5		0.81		0.50 to 1.30	0.38	
SRC		1.08		0.79 to 1.48	0.63	
**GSE17536 (OS)**						
CRK		1.14		0.49 to 2.66	0.75	
CXCL12		0.98		0.69 to 1.39	0.90	
ARPC5		1.27		0.37 to 4.35	0.70	
SRC		0.10		0.02 to 0.54	**0.01**	
**GSE29623 (OS)**						
CRK		0.83		0.15 to 4.52	0.83	
CXCL12		1.02		0.45 to 2.32	0.96	
ARPC5		0.25		0.03 to 2.03	0.19	
SRC		0.09		0.002 to 4.13	0.22	
**Multivariate analysis of EPHA2 genetic signature with hsa-miR-423-5p**						
		**Relative Risk**		**95% CI**	***P*** **value**	
**TCGA-COAD (OS)**						
ADAMTS14		1.07		0.97 to 1.18	0.18	
CREB1		0.98		0.87 to 1.1009	0.67	
hsa-miR-423-5p		1.00		0.99 to 1.00	0.69	
**GSE 29623 (OS)**						
ADAMTS14		0.35		0.04 to 3.02	0.34	
CREB1		0.82		0.13 to 5.12	0.83	
hsa-miR-423-5p		1.04		0.71 to 1.52	0.85	
**Multivariate analysis of EPHB2 genetic signature with hsa-miR-31-5p**						
		**Relative Risk**		**95% CI**	***P*** **value**	
**TCGA-COAD (OS)**						
CRK		0.97		0.93 to 1.01	0.11	
CXCL12		1.03		0.99 to 1.07	0.10	
ARPC5		0.99		0.97 to 1.02	0.65	
SRC		1.00		0.98 to 1.03	0.90	
hsa-miR-31-5p		1.00		1.00 to 1.01	**0.001**	
**GSE29623 (OS)**						
CRK		0.69		0.12 to 3.81	0.67	
CXCL12		1.31		0.53 to 3.21	0.56	
ARPC5		0.28		0.04 to 2.23	0.23	
SRC		0.12		0.003 to 4.88	0.26	
hsa-miR-31-5p		1.17		0.95 to 1.46	0.14	

Validation of the EphA2 and EphB2 signatures in GEO and TCGA-COAD public databases; Fold Change data of miR-423-5p and miRs-31 coherent target genes (miRNA and target gene expression levels are inversely correlated) with significant *P* values (*P* < 0.05) are indicated in bold **(a)**. Multivariate Cox proportional hazards regression analysis of EPHA2 and EPHB2 genetic signatures; significant *P* values (*P* < 0.05) are indicated in bold **(b)**

In addition, we evaluated the collective prognostic effect of the EphA2 and EphB2 signatures (multivariate Cox-regression analysis) (Table 1b). Regarding the EphA2 fingerprint, *ADAMTS14* was a risk factor for disease-free survival (DFS) (*P* < 0.001) and metastasis or recurrence within 3 years (MR3) (*P* < 0.05). Concerning the EphB2 fingerprint, *SRC* was a risk factor for overall survival (OS) (*P* < 0.01), *CXCL12* was significant for DFS (*P* < 0.01), *CRK, CXCL12* and *ARPC5* were significant for MR3 (*P* < 0.05). Furthermore, miR-31-5p was deemed a risk factor in TCGA-COAD (*P* < 0.001). Our results confirm the involvement of these molecules in a complex network affected by EphA2/EphB2 signaling pathways and their crosstalk.

## Conclusions

EphA2 and EphB2 contribute, in a pleiotropic manner, to the CRC pathogenesis. We demonstrated that the early dysregulation of EphB2 at the CRC onset correlates with specific stem-like properties and that the late overexpression of EphA2 triggers tumor progression signals in the invasive CRC phase. These properties are orchestrated by distinct miRNAomes and are associated with miR-31-5p/miR-31-3p, miR-423-5p and their EphA2/EphB2-related target genes. These signatures have significant clinical value and may unveil clinical predictive biomarkers and potential therapeutic markers.

## Additional files

Additional File 1: Supplementary Figures. (DOCX 3766 kb)

Additional File 2: Methods and Materials. (DOCX 24 kb)

Additional file 3: Supplementary Tables. (XLSX 77 kb)

## Abbreviations

ACF: Aberrant crypt foci
AOM: Azoxymethane
CRC: Colorectal cancer
CSCs: Cancer stem cells
DFS: Disease-free survival
DSS: Dextran sodium sulfate
GEO: Gene Expression Omnibus
ISCs: Intestinal stem cells
LD: Low dysplasia
miRNAs: microRNAs
MR3: Metastasis or recurrence within 3 years
OS: Overall survival
SD: Severe dysplasia
TCGA-COAD: The Cancer Genome Atlas - colon adenocarcinoma
TLDA: TaqMan Low-Density Array
