# Supplementary material for: EphB2 stem-related and EphA2 progression-related miRNA-based networks in progressive stages of CRC evolution: clinical significance and potential miRNA drivers
Source: Mol Cancer. 2018 Nov 30;17:169. doi: 10.1186/s12943-018-0912-z (PMC6271583; doi:10.1186/s12943-018-0912-z)
Supplement: Supplementary file 1 — Supplementary Figures. (DOCX 3766 kb) [file 12943_2018_912_MOESM1_ESM.docx]

**Additional File 1: Supplementary Figures**


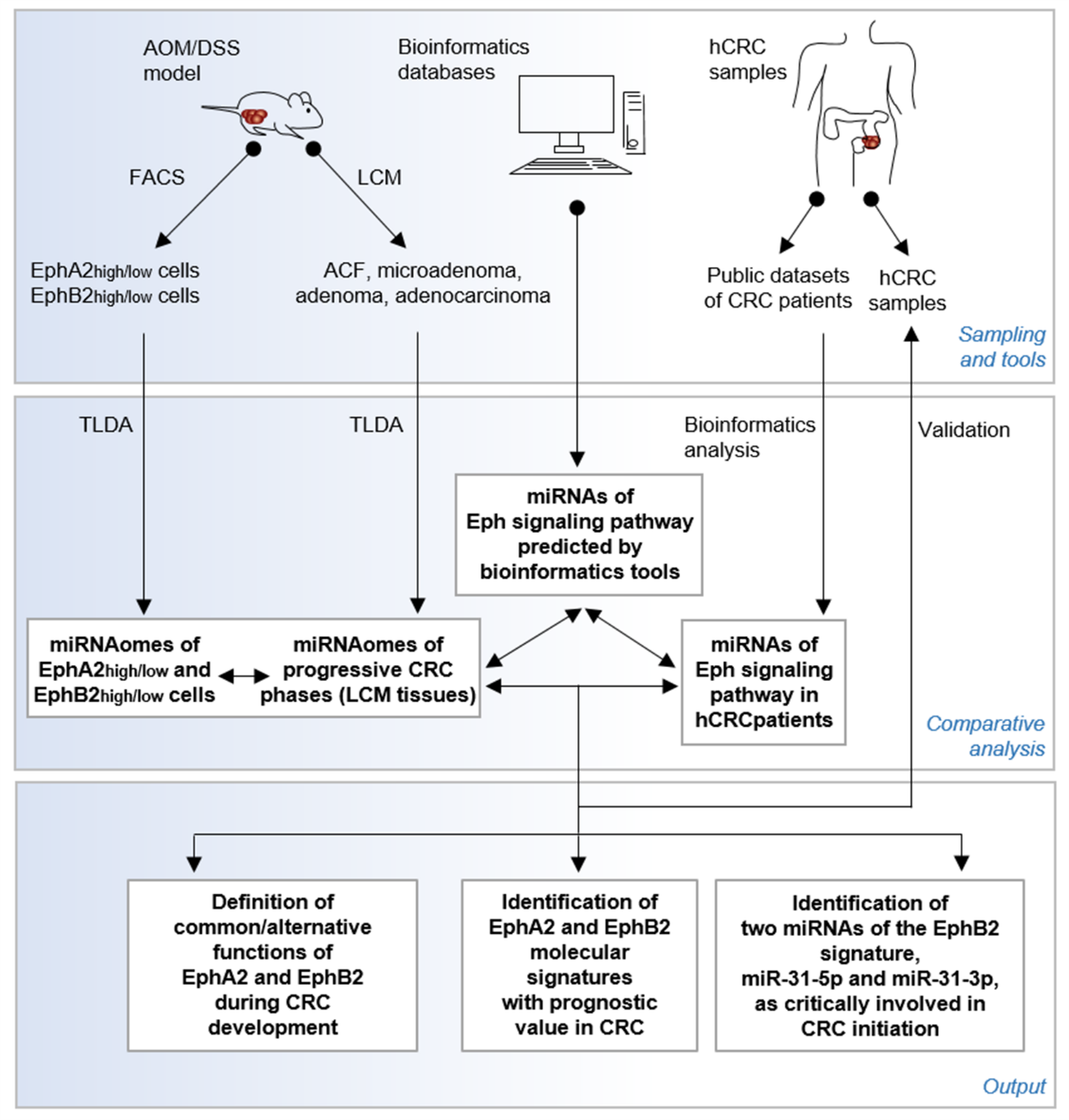


**Figure S1.** Workflow of the study: FACS-isolated CRC cells (EphA2_high/low_ and EphB2_high/low_) and LCM tissues were obtained from the AOM/DSS murine model to perform TLDA analysis and to identify the specific miRNAomes of distinct cells and tissues in different phases of CRC. Bioinformatics tools were used with public datasets (TCGA and GEO) containing CRC patient data to define Eph signaling pathway-related DE miRNAs in hCRC. These miRNA panels were compared with murine cell- and tissue-specific miRNAomes. The final outputs were the identification of i) common/alternative functions of EphA2/EphB2 during CRC development, ii) EphA2 and EphB2 molecular signatures with prognostic value in hCRC, and iii) the critical involvement of miR-31-5p and miR-31-3p (belonging to the EphB2 signature) in CRC initiation. Both hCRC tissues samples and public databases were used for the validation experiments. Abbreviations: CRC, colorectal cancer; DE, differentially expressed; FACS, fluorescence-activated cell sorter; hCRC, human colorectal cancer; LCM, laser capture microdissection; TLDA, TaqMan low-density array.

**
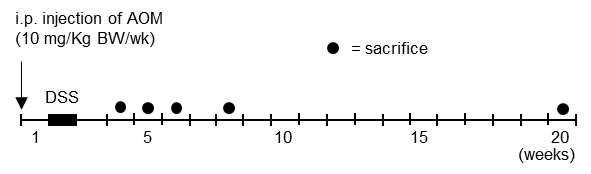
**

**Figure S2.** Schematic representation of the chemical-based protocol for the induction of murine CRC and time points of analysis. Abbreviations: AOM, azoxymethane; BW, body weight; DSS, dextran sodium sulfate; i.p. intraperitoneal; wk, week.

**
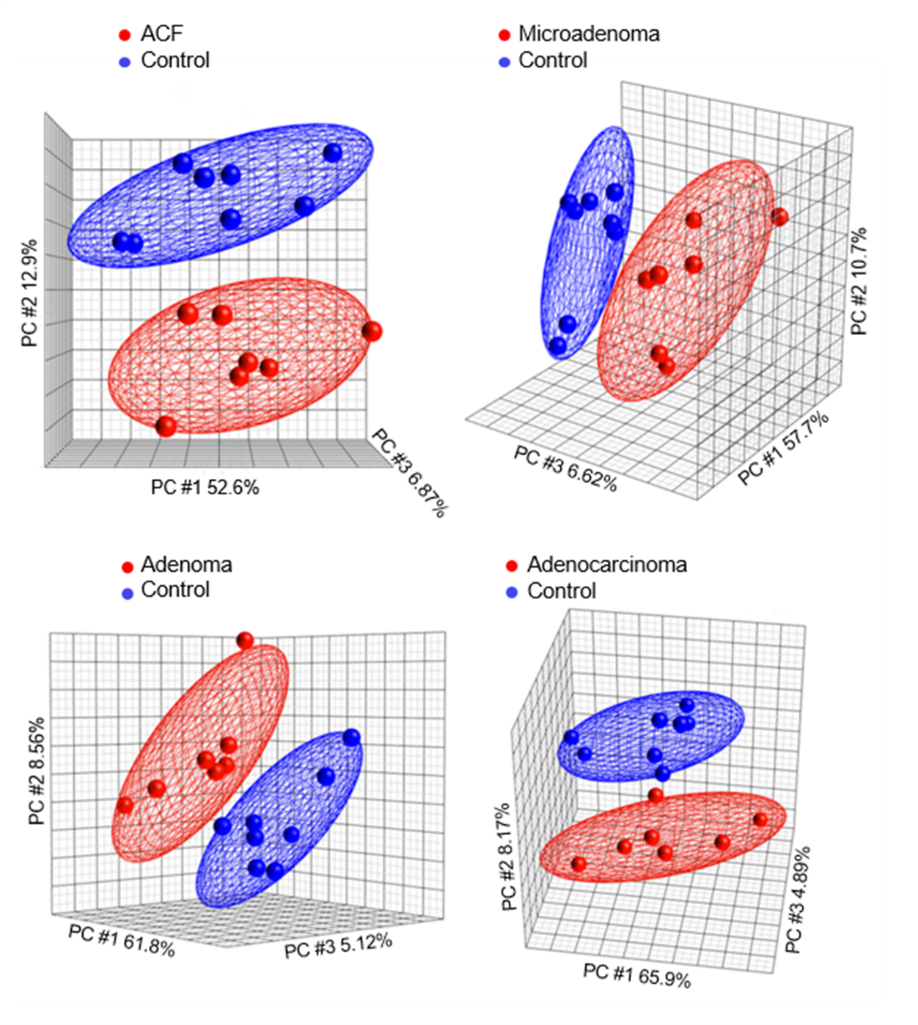
**

**Figure S3.** Principal Component Analysis (PCA) for each CRC phase (red) and Control samples (blue), belonging to the entire dataset obtained by two independent animal experiments, revealed a pattern of intra-class homogeneity and the separation of all the experimental groups. Control: normal colon mucosa of untreated mice.


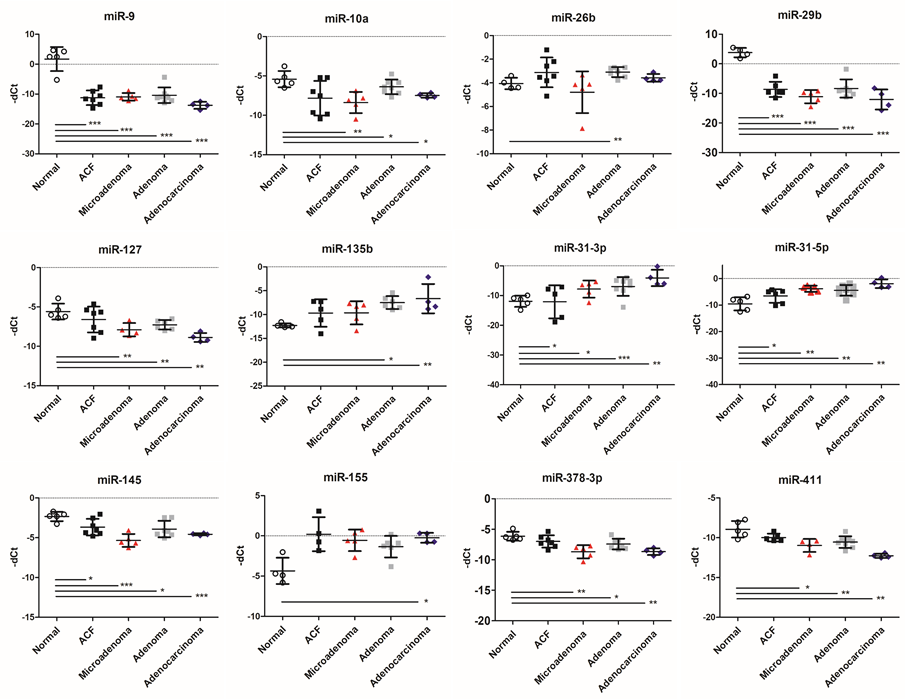


**Figure S4.** Multi-Gene qPCR validation of differential miRNAs expression on an independent cohort of 29 murine LCM tissues. MiRNAs expression levels were measured in five different conditions: Control (white circle), ACF (black square), Microadenoma (red square) Adenoma (grey square) and Adenocarcinoma (black rhombus). Control: normal colon mucosa of untreated mice. The results are expressed as –ΔCt values between the Ct value of the miRNA of interest and the geometric mean between Ct values of *U6 snRNA* and *snoRNA202*. Each dot represents the evaluation of the gene level in a single mouse. Statistically significant differences were calculated using Student’s *t*-test: **P* < 0.01, ***P* < 0.001, ****P* < 0.0001.

**
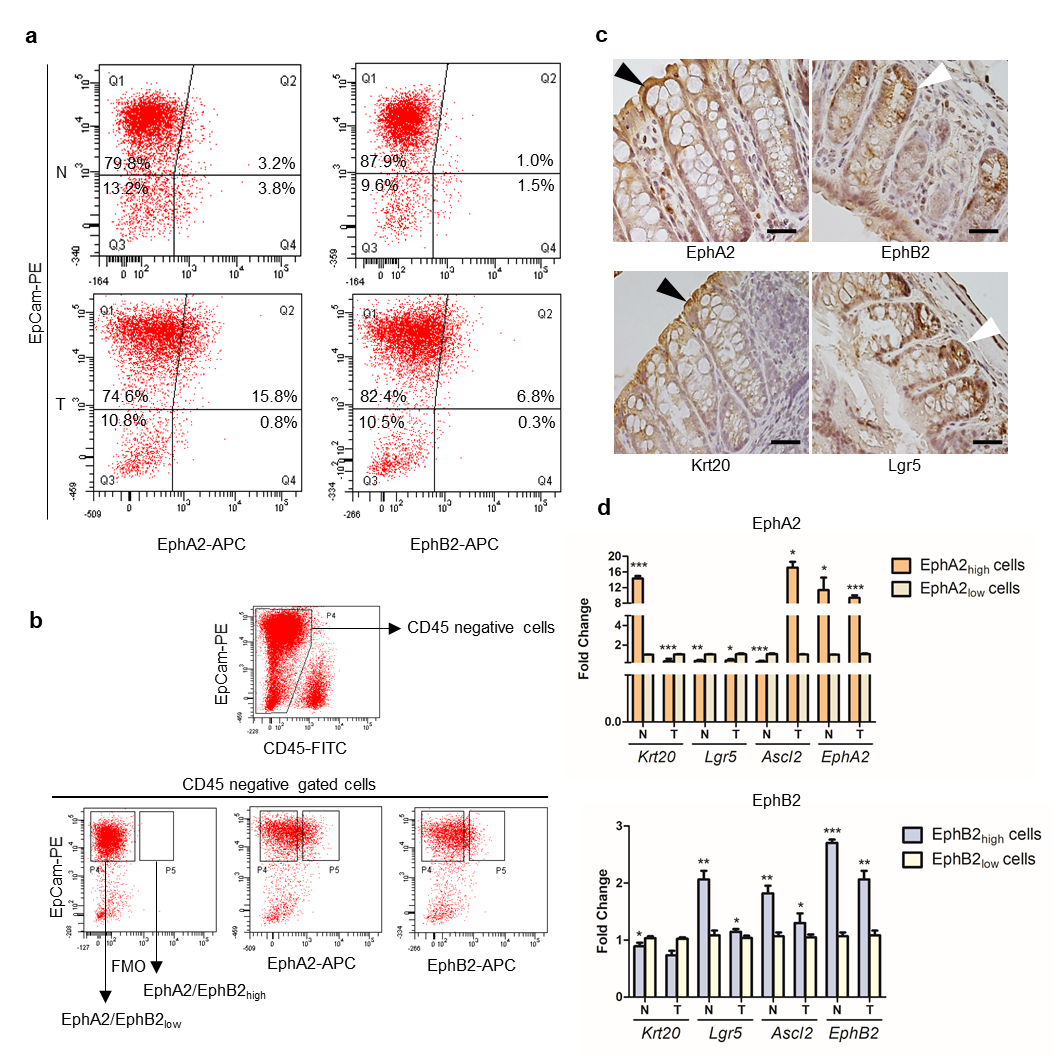
**

**Figure S5.** Isolation and characterization of EphA2_high/low_ and EphB2_high/low_ mouse colorectal cells. **a** Quantitative cytofluorimetric analysis of crypt cells stained for EphA2 revealed an increase of the EphA2_high_ cell subpopulation in colorectal tumor (T) with respect to normal mucosa (N). EphB2_high_ cells were poorly represented in both N and T. **b** FACS cell isolation. EphA2_high/low_ cells and EphB2_high/low_ cells were sorted after gating for CD45- and EpCAM+ staining to ensure epithelial identity. A fluorescence minus one (FMO) control stain strategy was used to precisely detect EphA2- and EphB2-expressing cells in the fully stained sample. **c** IHC analysis of the normal colorectal tissue of control untreated mice demonstrated maximum EphA2/Krt20 and EphB2/Lgr5 expression in the crypt apical columnar cells (black arrowhead) and basal crypt compartment (white arrowhead), respectively. Original magnification x20. Scale bar, 50 μm. **d** qPCR analysis of differentiation (*Krt20*) and stem cell markers (*Lgr5*, *Ascl2*) in EphA2_high/low_ and EphB2_high/low_ cell subpopulations purified from normal murine colon (N) and colorectal adenocarcinoma (T). Representative data of biological triplicates are shown. The geometric mean of *HPRT1* and *HMBS* was used for normalization. Data are represented as the means +/- standard deviation (SD). Student’s *t*-test, **P <* 0.01, ***P <* 0.001, ****P <* 0.0001.

**a. miR-31-5p and miR-31-3p**

| **Molecule Activity** | **Disease or Function** | **Expression evidence** | **Causal or Correlated** | **Findings** |
| --- | --- | --- | --- | --- |
| increased activity | Head and neck squamous cell carcinoma | upregulation | correlation | 1 |
| decreased activity | Endometrial cancer | downregulation | correlation | 2 |
| increased activity | Colony formation | not applicable | causal | 1 |
| decreased activity | Metastatic colorectal cancer | downregulation | correlation | 1 |
| decreased activity | Gastric cancer | downregulation | correlation | 2 |
| increased activity | Invasion of colorectal cancer cell lines | not applicable | causal | 4 |
| increased activity | Modification of colorectal cancer cell lines | not applicable | causal | 1 |
| increased activity | Migration of carcinoma cell lines, Migration of endometrial cancer cell lines | not applicable | causal | 1 |
| increased activity | Advanced cervical cancer | upregulation | correlation | 1 |
| increased activity | Cervical squamous cell carcinoma | upregulation | correlation | 1 |
| increased activity | Proliferation of lung cancer cells | not applicable | causal | 1 |
| increased activity | Migration of cells | not applicable | causal | 1 |
| decreased activity | Proliferation of lung cancer cells | not applicable | causal | 1 |
| decreased activity | Cell proliferation of colorectal cancer cell lines | not applicable | causal | 2 |
| increased activity | Migration of endothelial cells | not applicable | causal | 1 |
| increased activity | Pancreatic ductal adenocarcinoma | upregulation | correlation | 1 |
| increased activity | Colorectal cancer | upregulation | correlation | 1 |
| increased activity | Papillary thyroid carcinoma | upregulation | correlation | 2 |
| increased activity | Senescence of breast cancer cell lines | not applicable | causal | 2 |
| increased activity | Cell proliferation of squamous cell carcinoma cell lines | not applicable | causal | 1 |
| increased activity | Migration of squamous cell carcinoma cell lines | not applicable | causal | 1 |
| increased activity | Growth of tumor | not applicable | causal | 1 |
| decreased activity | Invasion of tumor cell lines | not applicable | causal | 2 |
| increased activity | Non-small cell lung carcinoma | upregulation | correlation | 1 |
| increased activity | Colorectal cancer | upregulation | correlation | 4 |
| decreased activity | Migration of colorectal cancer cell lines | not applicable | causal | 2 |
| increased activity | Cell movement of colorectal cancer cell lines | not applicable | causal | 4 |
| increased activity | Volume of tumor | not applicable | causal | 1 |
| increased activity | Stage II colorectal cancer | upregulation | correlation | 1 |
| decreased activity | Colorectal cancer | downregulation | correlation | 1 |
| increased activity | Invasion of carcinoma cell lines, Invasion of endometrial cancer cell lines | not applicable | causal | 1 |
| increased activity | Migration of tumor cell lines | upregulation | causal | 1 |
| increased activity | Mass of tumor | not applicable | causal | 1 |
| increased activity | Prostate cancer | upregulation | correlation | 1 |

**b. miR-423-5p**

| **Molecule Activity** | **Disease or Function** | **Expression evidence** | **Causal or Correlated** | **Findings** |
| --- | --- | --- | --- | --- |
| increased activity | Breast cancer | upregulation | correlation | 10 |
| increased activity | Gene silencing | not applicable | causal | 1 |
| decreased activity | Rapidly progressive idiopathic pulmonary fibrosis | downregulation | correlation | 1 |
| increased activity | Alzheimer disease | upregulation | correlation | 2 |
| increased activity | Nonobstructive azoospermia | upregulation | correlation | 1 |
| increased activity | Rapidly progressive idiopathic pulmonary fibrosis | upregulation | correlation | 1 |
| increased activity | Failure of heart | upregulation | correlation | 1 |
| decreased activity | Class II lupus nephritis | downregulation | correlation | 1 |
| decreased activity | HER2 negative hormone receptor negative breast cancer | downregulation | correlation | 1 |
| increased activity | Endometrial cancer | upregulation | correlation | 1 |
| unknown change in activity | Esophageal cancer | not applicable | correlation | 1 |
| increased activity | Colorectal cancer | upregulation | correlation | 1 |

**Figure S6.** Functional analysis of **a** miRs-31 and **b** miR-423-5p using the BioProfiler tool of Ingenuity Pathway Analysis. We found that miR-423-5p was associated to a few types of cancers, including colorectal cancer.  Similarly, miR-31 was correlated to colorectal cancer. Molecule Activity column reports the overall activity of the molecule in the particular finding. "Disease or Function" shows a list of the disease or function associated with the molecule. "Expression Evidence" reports the literature evidence that a gene is over or under-expressed in cells or tissues experiencing a particular disease or function. "Causal or Correlated" column is "Causal" if literature implicates that molecule as driving a disease or function, or if it is an OMIM or Jax finding correlation; "Not causal" if literature, GWAS study, COSMIC finding, biomarker findings indicate only that an association was observed or predicted. "Findings" counts the number of findings that support that row of the table.

**
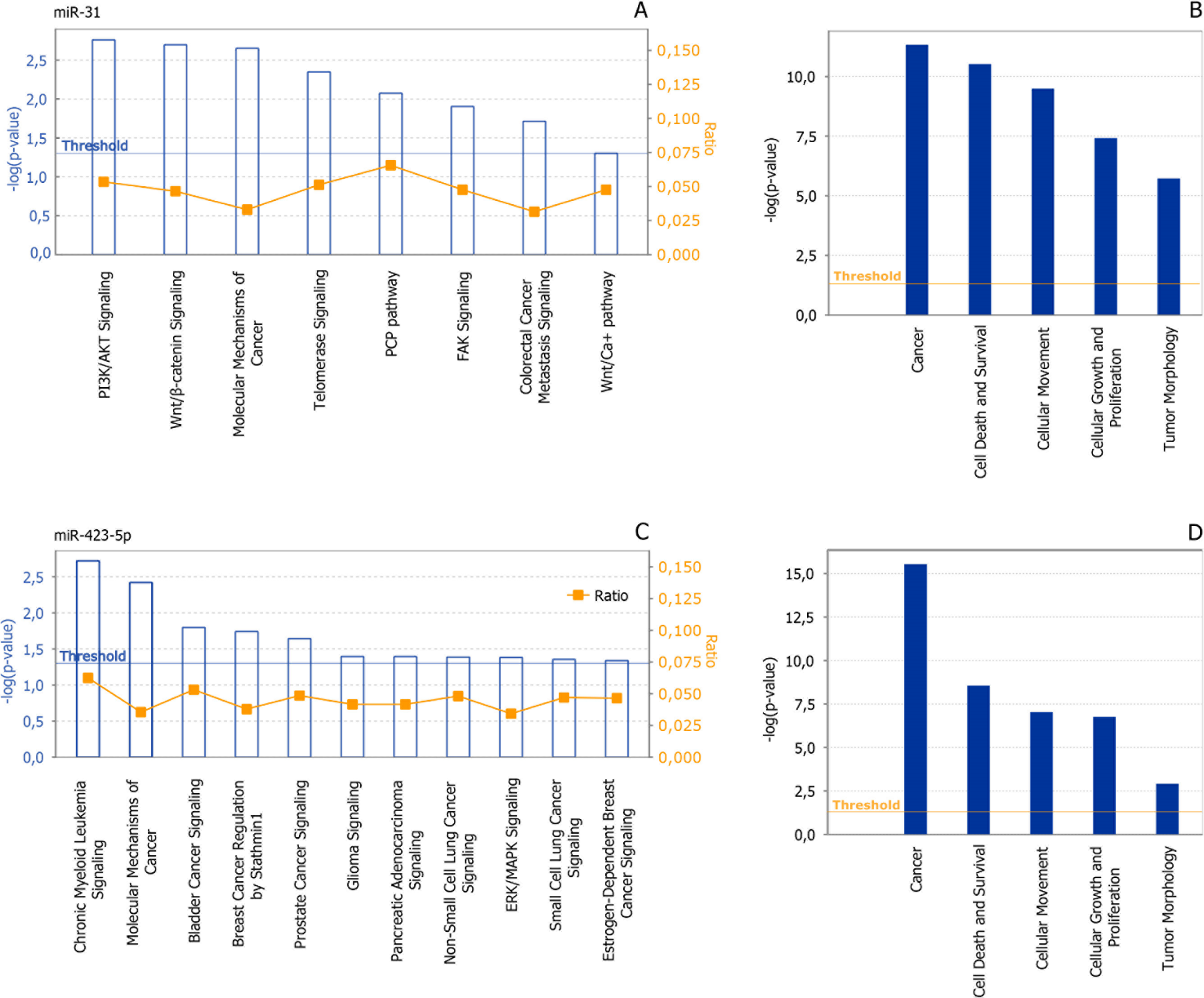
**

**Figure S7.** Statistically significant cancer-related pathways enriched by the genes target of **a** miR-31-5p and miR-31-3p and **c** miR-423-5p, sorted by *P* value (-Log, bars). The ratio of genes found in each pathway over the total number of genes in that pathway is plotted as orange squares. Cancer-related enriched biological functions and phenotypes by genes target of **b** miR-31-5p and miR-31-3p and **d** miR-423-5p, sorted by *P* value (-Log). The threshold lines correspond to a *P* value of 0.05. Statistical significance was calculated using Fisher’s exact test as implemented in IPA.

**
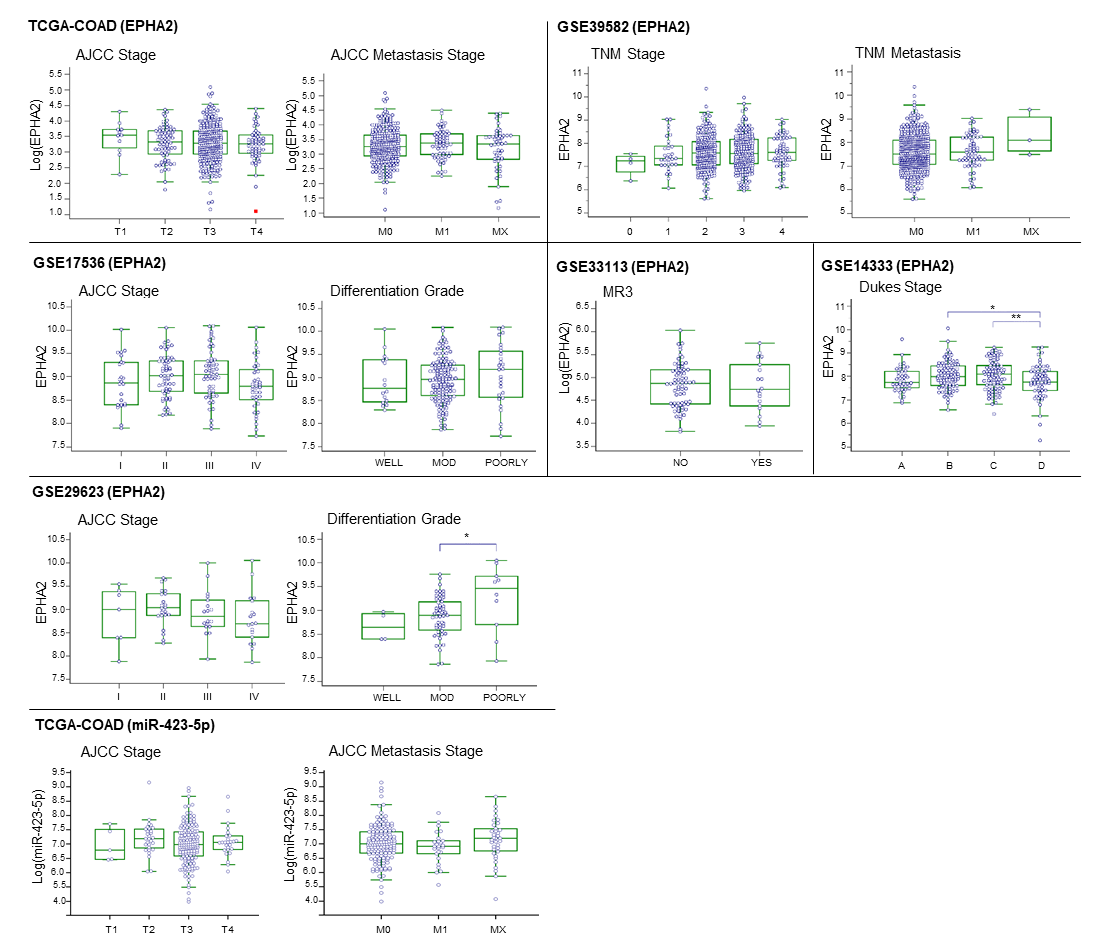
**

**Figure S8.** EphA2 and miR-423-5p analysis on hCRC. No significant changes are observed in the expression levels of both EphA2 and miR-423-5p with tumor staging progression (AJCC stage, TNM stage, Dukes stage), metastasis, and differentiation grade. Dots indicate individual data points, and their distribution is represented by violin and box plots. The box plots show the interquartile range and median value of the distribution. Abbreviations: FC, fold change; MOD, moderately differentiated; MR3, metastasis or recurrence within 3 years. **P <*0.05; ***P <*0.01; Mann-Whitney-U test.

**
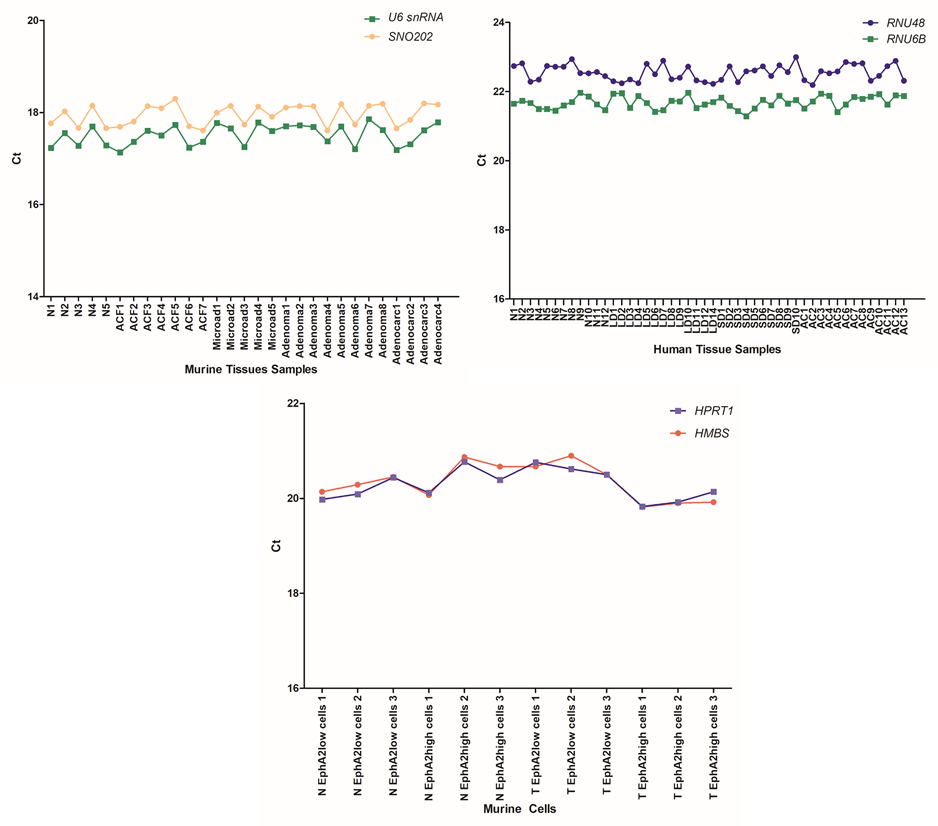
**

**Figure S9.** Expression profile of endogenous controls used across murine Laser-Capture microdissected tissues (*U6 snRNA* and *sno202*), human FFPE tissues (*RNU48* and *RNU6B*) and murine sorted cells (*HPRT1* and *HMBS*). Abbreviations: N, normal colon mucosa; ACF, aberrant crypt foci; Microad, microadenoma; Adenocarc, adenocarcinoma; LD, low dysplasia adenoma; SD, severe dysplasia adenoma; AC, adenocarcinoma; N, normal colon mucosa of untreated mice; T, colorectal adenocarcinoma.
