# Supplementary material for: EphB2 stem-related and EphA2 progression-related miRNA-based networks in progressive stages of CRC evolution: clinical significance and potential miRNA drivers
Source: Mol Cancer. 2018 Nov 30;17:169. doi: 10.1186/s12943-018-0912-z (PMC6271583; doi:10.1186/s12943-018-0912-z)
Supplement: Supplementary file 2 — Methods and Materials. (DOCX 24 kb) [file 12943_2018_912_MOESM2_ESM.docx]

**Additional File 2: Methods and Materials**

**Animals and sample processing**

Two sets of 50 6-week-old BALB/c mice were used to reproduce the azoxymethane (AOM)/dextran sodium sulfate (DSS) model of CRC. The study was approved by the Italian Ministry of Health with decree no. 336/2013-B in accordance with the institutional guidelines for laboratory animal care and adhered to ethical standards.

For each experiment, mice were treated as previously described [1]. Five AOM/DSS-treated and five untreated (control) mice were sacrificed at consecutive time points to analyze the progressive CRC stages, i.e. ACF and microadenoma (4th and 5th weeks), adenoma (6th and 8th weeks) and adenocarcinoma (20th week). The large intestine was removed and processed as previously described [2]. Colon mucosa or tumors were excised and then stored at -80 °C until RNA extraction. Other areas of the large intestine were fixed in formalin, embedded in paraffin and then stored for histological examination.

**Laser capture microdissection (LCM) of murine tissues**

Distinct formalin-fixed paraffin-embedded (FFPE) sections (12 μm) of colorectal normal mucosa and adenocarcinoma samples were affixed to frame slides for membrane-based LCM. Slides were washed in xylene to remove paraffin, rehydrated by a graded ethanol series and stained with hematoxylin. Samples of interest were microdissected using Leica LMD6000 Microsystems (Leica). Captured samples were resuspended in TRIzol® Reagent and stored at -80 °C until processing.

**Histopathological analysis of murine tissues**

FFPE sections of murine and human colorectal samples were stained using hematoxylin and eosin according to standard procedures.

Murine ACF, microadenoma, adenoma and adenocarcinoma were diagnosed according to the previously defined histopathological criteria [3]. Immunohistochemistry (IHC) was performed on murine FFPE sections after antigen retrieval in sodium citrate. The samples were stained with rabbit anti-mouse EphA2 or EphB2 (LSBio, LS-B1794, LS-A2897, 1:200), goat anti-mouse Krt20 or anti-mouse Lgr5 (Santa Cruz, N-13, C-16, 1:100) antibodies overnight at 4 °C. The image data were analyzed using NIS FreeWare 2.10 software (Nikon).

**Isolation of murine EphA2 and EphB2 cells**

Adenocarcinoma and normal mucosa were removed from mice and directly processed for a FACS-assisted cell isolation using the protocol described elsewhere [4]. The following mixture of anti-mouse primary antibodies was used to stain up to 10^7^ cells: CD45-FITC, EpCAM-PE (eBioscience, Mab 30-F11, Mab G8.8), EphA2-APC, EphB2-APC (R&D Systems, Mab 233720, Mab 512012) or appropriate isotype controls. The fixable viability dye eFluor 780 (eBioscience) was added to identify and discard dead cells and debris. CD45-EpCAM+EphA2_high/low_ and CD45-EpCAM+EphB2_high/low_ normal and tumor cells were sorted in a FACSAria I (BD Biosciences), using BD FacsDIVA software version 6.1.3. A Fluorescence Minus One (FMO) control strategy allowed the accurate identification of positively stained cells in the fully stained sample [5] (Additional File 2: Fig. S5b). After collection, sorted cells were resuspended in TRIzol Reagent® and stored at -80 °C. Validation of cell subpopulation identities was achieved by quantitative Real-Time PCR (qPCR) to determine the gene expression levels of *EphA2, EphB2* and stemness/differentiation genes.

**CRC patient samples**

Colonic mucosa tissues from 49 patients, including adenocarcinoma (n = 13), adenoma with high-grade dysplasia (n = 10), adenoma with low-grade dysplasia (n = 14) and non-tumoral tissues (n = 12), were obtained in collaboration with the IRCCS “Regina Elena” National Cancer Institute, Rome, Italy. All patients signed an informed consent in accordance with the WMA Declaration of Helsinki 2013 and approved by the Ethics Committee of the IRCCS “Regina Elena” National Cancer Institute. All patients with adenocarcinoma were staged by TNM guidelines. Tumor tissue and distal normal mucosa were isolated from patients during surgical resection, fixed in formalin and embedded in paraffin.

With the support of an expert pathologist, FFPE human samples stained with hematoxylin and eosin were selected by stage based on the following histological criteria: i) adenoma with low dysplasia (LD-adenoma) – non-complex crypts with regular, elongated nuclei, pseudo- or partially stratified; parallel crypts (pretumoral lesion); ii) adenoma with severe dysplasia (SD-adenoma) – marked pseudostratification or stratification of neoplastic cells, with atypical mitoses and marked loss of polarity; back-to-back and cribriform glands; iii) adenocarcinoma – irregular and complex glands, an increased nucleus to cytoplasm ratio, marked loss of polarity and desmoplasia.

**RNA extraction and qPCR**

Total RNA was isolated from LCM-murine/FFPE-human tissues and murine cells using TRIzol® Reagent according to the manufacturer’s instructions. RNA was retrotranscribed using a High-Capacity cDNA Reverse Transcription kit (Applied Biosystems) and qPCRs were performed using the following TaqMan® assays (Applied Biosystems): LGR5 (Mm00438890_m1), ASCL2 (Mm01268891_g1), KRT20 (Mm00508106_m1), HPRT1 (Mm00446968_m1), HMBS (Mm01143545_m1), mmu-miR-9 (000583), mmu-miR-10a (000387), mmu-miR-26b (000407), mmu-miR-29b (000413), mmu-miR-127 (000452), mmu-miR-145 (002278), mmu-miR-378-3p (002243), mmu-miR-411 (001610), mmu-miR-135b (002261), mmu-miR-31-5p (000185), mmu-miR-31-3p (002495), mmu-miR-155 (002571), snoRNA202 (001232), U6 snRNA (001973), hsa-miR-31-5p (002279 ), hsa-miR-31-3p (002113), RNU48 (001006), RNU6B (001093).

Data were analyzed by ABI 7900HT SDS software 2.3 (Applied Biosystems). Relative expression was calculated according to the 2^-ΔΔCt^ method. Normalization was achieved by geometric mean of *HPRT1* and *HMBS* for gene expression data, *U6 snRNA* and *snoRNA202* for murine miRNAs and *RNU48* and *RNU6S* for human miRNAs (Additional file 2: Figure S9). All experiments were performed in biological triplicates. qPCR results were analyzed by Student’s *t*-test using Prism 3.0 GraphPad Software.

**TaqMan miRNA low-density arrays**

The TaqMan® miRNA Low-Density Arrays (TLDAs) (Applied Biosystems) were used for the miRNA expression profiling of murine samples. Biological quadruplicates of LCM tissues were analyzed in two main analytical runs (two independent animal experiments), whereas biological duplicates of FACS-sorted cells were analyzed. Total RNA was retrotranscribed and the cDNA products were loaded onto TaqMan® array Rodent microRNA A+B Cards Set version 3.0 to quantify up to 641 rodent miRNAs. Quantitative miRNA expression data were acquired using ABI 7900HT SDS software.

Since two main analytical runs were performed on LCM tissues, any unwanted batch effect was mitigated by the Batch Effect removal tool of Partek^®^ Genomic Suite^®^ software (version 6.6; 2016). The effectiveness of this strategy was assessed by principal component analysis (PCA). ΔCt values were calculated after normalization against a pool of housekeeping genes (HKGs) using the SLqPCR R package (<https://www.bioconductor.org/packages/release/bioc/html/SLqPCR.html>) [6]. *U6 snRNA* and *snoRNA202* were the most stable HKGs. Their expression levels were averaged geometrically before being subtracted from Ct values. A subset of four normal samples was used as inter-run calibrators. Furthermore, a sparse p least square-discriminant analysis (sPLS-DA) was conducted through the *mixOmics* R package (<http://mixomics.org>) on the entire dataset to detect collective variations among the CRC phases and controls. The raw data for TLDAs were analyzed by calculating ΔCt expression values as the difference between the Ct value of each miRNA and the geometric mean of the Ct values of *U6 snRNA* HKG.

Fold Change (FC) expression values were calculated with the ΔΔCt method. *P* values were calculated by two-tailed normal distribution or two-tailed Student’s *t*-test, when appropriate. Type I errors were decreased by applying the Benjamini-Hochberg procedure to all raw *P* values. MiRNAs exhibiting a *P* value ≤ 0.05 and an absolute value of ΔΔCt ≥ 2 were considered DE.

**Functional enrichment analysis**

Functional enrichment analyses of DE miRNAs and EphA2/EphB2 specific-signatures were performed by Ingenuity Pathway Analysis (IPA; QIAGEN). Experimentally verified target genes were retrieved from miRWalk 3.0 (http://zmf.umm.uni-heidelberg.de/apps/zmf/mirwalk/) and miRTarBase 7.0 (http://mirtarbase.mbc.nctu.edu.tw). The functional enrichment analysis was based on the prior calculation of the Z-scores, by which we inferred the activation states of the obtained biological functions. This was possible when *P* values and FC values accompanied the DE miRNA lists submitted to IPA. An enrichment score (Fisher’s exact test, “overlap *P* value”) was calculated to measure the likelihood that an association between a set of miRNAs and a related function was not due to the chance. We considered *P* values < 0.05 and Z-scores > 0 (activation) or < 0 (inhibition).

**CRC patient databases and bioinformatics analysis**

Genes and miRNAs of interest were validated in a multi-cohort study of public human CRC expression profiles (total n = 1,663). Nine CRC cohorts (Additional file 3: Table S6) were collected from the Gene Expression Omnibus (GEO) public data repository and from The Cancer Genome Atlas-Colon Adenocarcinoma dataset (TCGA-COAD), using the *GEOquery* R package [7] and the NCI Genomic Data Commons (GDC) Data Portal [8], respectively.

Cohort 5, cohort 8 and TCGA-COAD allowed the analysis of the overall survival (OS) of patients, i.e., the time from study enrollment to death. Cohort 4 allowed the analysis of disease-free survival (DFS), which is the difference between the time of surgery and the time of the first death or cancer recurrence. For cohort 7, we considered the time to metastasis or recurrence within 3 years (MR3) of the CRC.

The association between EphB2 expression and the clinical characteristics of individuals was tested through the two-tailed Mann-Whitney-U test. DE miRNAs and genes were obtained through one-way analysis of variance (ANOVA) performed with Partek^®^. Survival analysis was performed with the *maxstat* R package (<https://CRAN.R-project.org/package=maxstat>). The Maximally Selected Rank Statistics (maxstat) was used to determine the optimal cutpoint of continuous variables (i.e., gene expression values) which best splits individuals into high and low EphA2/EphB2. Prognostic significance was estimated by the log-rank test and represented as Kaplan-Meier curves. Multivariate Cox proportional hazards regression analysis was used to evaluate the impact of EphA2- and EphB2-specific signatures on survival, independently of other clinical parameters. *P* values < 0.05 were considered statistically significant.

**References**

1. De Robertis M, Massi E, Poeta ML, Carotti S, Morini S, Cecchetelli L, et al. The AOM/DSS murine model for the study of colon carcinogenesis: From pathways to diagnosis and therapy studies. J Carcinog 2011;10:9.
2. Merlos-Suárez A, Barriga FM, Jung P, Iglesias M, Céspedes MV, Rossell D, et al. The intestinal stem cell signature identifies colorectal cancer stem cells and predicts disease relapse. Cell Stem Cell 2011;8:511–24.
3. Boivin GP, Washington K, Yang K, Ward JM, Pretlow TP, Russell R, et al. Pathology of mouse models of intestinal cancer: consensus report and recommendations. Gastroenterology 2003;124:762–77.
4. De Robertis M, Loiacono L, Fusilli C, Poeta ML, Mazza T, Sanchez M, et al. Dysregulation of EGFR Pathway in EphA2 Cell Subpopulation Significantly Associates with Poor Prognosis in Colorectal Cancer. Clin Cancer Res 2017;23:159–70.
5. Roederer M. Spectral compensation for flow cytometry: visualization artifacts, limitations, and caveats. Cytometry 2001;45:194–205.
6. Vandesompele J, De Preter K, Pattyn F, Poppe B, Van Roy N, De Paepe A, et al. Accurate normalization of real-time quantitative RT-PCR data by geometric averaging of multiple internal control genes. Genome Biol 2002;3: RESEARCH0034 (202).
7. Davis S, Meltzer P. GEOquery: a bridge between the Gene Expression Omnibus (GEO) and BioConductor. Bioinformatics 2007;14:1846–7.
8. Grossman RL, Heath AP, Ferretti V, Varmus HE, Lowy DR, Kibbe WA, et al. Toward a Shared Vision for Cancer Genomic Data. N Engl J Med 2016;375:1109–12.
